# Characterization of Toll-Like Receptor-4 (TLR-4) in the Spleen and Thymus of Swiss Albino Mice and Its Modulation in Experimental Endotoxemia

**DOI:** 10.1155/2015/137981

**Published:** 2015-02-05

**Authors:** Chandrayee Ghosh, Biswadev Bishayi

**Affiliations:** Department of Physiology, Immunology Laboratory, University of Calcutta, University Colleges of Science and Technology, 92 APC Road, Kolkata, West Bengal 700009, India

## Abstract

Expression of innate immune receptors varies among organs and species and within different strains among the same species; thus, periodic classification of different pattern recognition receptors in the available strains is necessary to initiate different therapeutic approaches to combat inflammation. On characterization of TLR-4 in spleen and thymus of Swiss albino mice—with no reports of TLR-4 expression—induced with endotoxemia, it was found that the mode of expression varied among the organs at both mRNA and protein level in a time-dependent manner. Their functionality was verified by measuring proinflammatory and anti-inflammatory cytokines. In the *in vitro* study using isolated macrophages and lymphocytes from the same organs, the expression of TLR-4 after a shorter period of LPS stimulation was verified. The results substantiated the potent role of macrophage on LPS challenge compared to lymphocytes. The diverse pattern of TLR-4 expression on different cell population indicated their distinct functional activity in LPS-endotoxemia. It may be hypothesized that the expression patterns of TLR-4 could be different based on the anatomical localization and the varying bacterial milieu or bacterial endotoxin encountered in each anatomical location. Thus, blocking TLR-4 or administering IL-6 or IL-10 might impart protection against endotoxemia in the clinical field.

## 1. Introduction

Endotoxemia in response to bacterial lipopolysaccharide (LPS) is characterized by the production of inflammatory cytokines such as tumor necrosis factor alpha (TNF-*α*), interleukin 1 (IL-1), and gamma interferon (IFN-*γ*) along with the release of highly reactive oxygen and nitrogen intermediates which are thought to contribute significantly to the end stage tissue damage in this disease [1–3]. Injection of LPS, the major component of the outer cell wall of Gram-negative bacteria, into the bloodstream showed pathophysiological changes that were similar to those seen in sepsis in experimental animals [4] as well as human volunteers [5]. Bacterial LPS in the bloodstream induced the over expression of a large amount of inflammatory mediators in the body, which had been thought to contribute to the LPS-induced symptoms of septic shock and mortality [6]. Sepsis has been described as a complex clinical syndrome that could result from a detrimental and dysregulated host response to bacterial infection [7, 8]. It has also been emphasized that the mortality rates of patients suffering from endotoxin induced sepsis [7] was quite alarming even after provided with antibiotics and the best available supportive care. High frequency of septic shock caused mortality and the supposed modalities that work in an experimental setting do not always find success in clinical practice. These existing challenges reinforced and fortified the need for new animal models, strains, and dose and time-dependent studies to reproduce the various pathophysiological changes associated with septic shock and identify new therapeutic strategies. Previous studies have manifested the role of Toll-like receptors (TLRs) in various pathological conditions along with sepsis. Endotoxin or lipopolysaccharide of Gram-negative bacteria being a major causative agent of sepsis as well as an established ligand of Toll-like receptor (TLR-4) [9–11] endorsed us to characterize in detail this receptor and its modulation on LPS administration in Swiss albino strain of mice as no such reports on this strain has been found till date.

As mouse has been considered as a prime organism of choice for modeling human diseases, many inbred strains of mice have been described providing a wealth of different genotypic and phenotypic information for genetic and other studies. As new strains are generated and others become extinct, it is considered to be useful to review and characterize periodically, what strains are available and how are they related to the others [12, 13]. Thus, this study was aimed to characterize the expression of TLR-4 in the lymphoid organs of Swiss albino mice and also its cellular response in an* in vitro* setting. The presence of TLR-2 and its response to* Staphylococcus aureus* for a time interval of 3, 9, and 15 days after infection in spleen, thymus, and lymph node has been reported by us recently [14]. In this study, the work was continued on the lymphoid organs concentrating on a different member of the TLR family.

TLRs are transmembrane proteins of the interleukin- (IL-) 1 receptor superfamily which are able to recognize PAMPs and mediate the production of cytokines necessary for the development of effective immunity [15]. Upon stimulation, TLRs recruit IL1R1-associated protein kinases mediated by MYD88 and induce the activation of nuclear factor-*κ*B (NF-*κ*B) and mitogen-activated protein kinases (MAPK), leading to cytokine induced inflammation [16] and thus the development of antigen-specific adaptive immunity. Eleven members of this family have been identified so far and individually these receptors comprise a group of evolutionary conserved pattern recognition molecules that has the ability to recognize distinct PAMPs. Few years ago it was suggested that several TLR orthologues were not only expressed differently among different species like mice and humans but also intraindividual and intraspecific variation in the expression of TLR transcripts in various cell types exists. Moreover, the transcription regulation on cellular activation were also found to differ thus encouraging studies in new strain of mice as already mentioned before [17].

TLR-4 was the first mammalian homology of* Drosophila* toll to be discovered and is the most critical sensor for the recognition of LPS. It can regulate the innate immune response by triggering signal transduction pathways associated with it [18]; therefore, it is always critical to understand the expression of these proteins for a better comprehension of the host response to pathogens. As mentioned earlier, recent evidences have accumulated the significance of TLRs in various pathological conditions such as sepsis and inflammation in various organs under different environmental conditions. Toll-like receptor-4 was found to be upregulated during intestinal inflammation [19], surgical stress [20] and obstructive jaundice [21]. TLR-4 involvement in vascular organ maladies such as intestinal colitis [22], myocardial inflammation [23], kidney [24], and injured and alcoholic liver [25] has also been found. Receptors were reported to be widely distributed, not only in immune cells, such as macrophages [26] and dendritic cells [27] but also in the epithelia of the respiratory [28], digestive [29], and urinary tracts [30]. From these reports it could be deduced that the differential expression patterns of TLR-4 during different pathogenesis may reflect their anatomical localization and cellular/organ exposure to microbial challenge [31].

TLR-4 is responsible for recognition of LPS and this was proved earlier from studies on the LPS-hyposensitive phenotype of the C3H/HeJ mouse as well as in C57BL/10ScCr mice [10, 11]. Studies on wild type C57BL/6 mice by Ehrentraut et al. later confirmed the finding [32] but contradictory results were reported by Matsuguchi et al. stating that at mRNA level TLR-4 on macrophages of lymphoid organs from BALB/c mice were unresponsive to LPS as compared to TLR-2 [33]. Hence, a strainwise variation was indicated in prior studies as well. Another important fact in this regard could be the existing divergence in the LPS structure among Gram-negative bacteria and it may be reasonable to presume that TLR-4 respond to certain types of LPS better that TLR-2 while TLR-2 respond better to others [34] resulting in different pathophysiology of the resultant endotoxemia. Moreover, while studying the expression of TLR-4 an interesting fact surfaced about its expression in human and murine cells in certain experiments. On stimulation with LPS, the expression of TLR-4 was found to be increasing in human monocytes and/or macrophages [35] but was being downregulated in murine macrophages on LPS activation [33]. In support, it was suggested that sometimes posttranscriptional destabilization of murine TLR-4 mRNA after LPS administration might be responsible for the latter observation [36]. Changes in mRNA stability have not been reported for any other TLR genes so far. Thus, TLR-4 expression should be studied both at mRNA and proteins levels to clarify whether individual variations among species extend to the level of post transcriptional or translational regulation.

TLR-4 can recognize molecular patterns associated with a wide range of microbial pathogens in order to initiate transcription of various proinflammatory cytokines such as IL-1*β*, TNF-*α*, IL-, and IL-8 [37] and also other proinflammatory proteins such as inducible NO synthase (iNOS) and acute phase response protein like CRP (C-reactive protein) and SAP (Serum amyloid P-component). CRP is a component of the acute phase response and its serum level could increase to varying degree in response to infection, inflammation, and trauma [18]; thus, it could be used as an inflammatory marker in mouse model of endotoxin induced inflammation by* E. coli* [38, 39]. CRP has been validated as a good surrogate marker of disease severity and could be correlated with the final outcome of endotoxemia [40]. Another important fact is that CRP has its half-life measured in days and is not subjected to circadian rhythm. These factors reduced the variabilities while measuring CRP and contribute to its success as a surrogate marker.

Since the innate immune system has been thought to operate mainly via TLRs on macrophages, it is conceivable that expression of TLRs on macrophages and their responsiveness to the agonists could be of great importance for inflammatory response and host defense mechanism in LPS induced endotoxemia. However, they have not yet been examined in Swiss albino mice. This study aimed at investigating the expression of TLR-4 on macrophages and their capacity of TLR-mediated cytokine production in LPS challenged Swiss albino mouse.

In this study, an experimental endotoxemic model of Swiss albino mice was used to observe the expression and functionality of TLR-4 in spleen and thymus which was complemented with an* in vitro* study on macrophages and lymphocytes isolated from these lymphoid organs. While there are many reasons to believe that* in vivo* studies have the potential to offer conclusive insights about the nature of medicine and disease, but there are a number of ways in which the conclusions could be misleading. On the other hand, an* in vitro *experiment fails to replicate the precise cellular conditions of an organism and sometimes may lead to results that do not correspond to the circumstances occurring around a living organism. Thus, this study was an initial stage to correlate these two experimental conditions and authenticate the finding as far as possible to get a much better insight of the mode of action of the immune system during experimental endotoxemia.

## 2. Materials and Methods

### 2.1. Material

The LPS (obtained from* E. coli* O55: B5) was bought from Sigma Chemicals (cat no- L2880, this lyophilized powder of LPS was of Premium quality, purified by phenol extraction, containing only <3% impurities as estimated by Lowry method). The antibodies and substrate required for Western Blot were purchased from Abcam, the ELISA kits for cytokine assays were purchased from RayBiotech, Inc., and the CRP kit was bought from My BioSource. All other chemicals were of analytical grade.

### 2.2. Animal

Male Swiss albino mice, 6–12 weeks of age and weighing 20 ± 4 g, were obtained from Chittaranjan National Cancer Institution, Kolkata, West Bengal, and immediately randomized in plastic cages with filter bonnets and saw dust bedding. Six mice were housed per cage with food and water* ad libitum* and were kept in quarantine for 8 days. Animals were maintained throughout at a temperature of about 21–24°C, 40–60% humidity and a 12 hours light dark cycle. Animals were fed with normal rodent diets. All experiments performed in this study were approved by the Institutional Animal Ethical Committee (IAEC) [Phy/IAEC/proposal/BB-2/2012 dated-02.01.2012] as per guidelines of the CPCSEA, Ministry of Environment and Forests, Government of India.

### 2.3. Treatment with LPS* In Vivo*

Mice were injected with LPS intraperitoneally (I.P) at a dose of (5 *μ*g/20 gm of body weight) which was previously standardized in our laboratory after monitoring the mortality rate [41]. Control mice were injected only with sterile saline. Treatment with 5 *μ*g/mouse by I.P injection induced typical symptoms of inflammation by 2–6 hours after treatment as indicated by reduced activity, ruffled fur, and shivering. These effects were less evident after administration of lower dose of LPS. The mice challenged with LPS were euthanized painlessly at 3, 6, 12, 24, 48, and 72 hours after treatment.

### 2.4. Collection of Tissues and Blood Then Preparation of Serum

Prior to blood collection, the animals were weighed and then anesthetized by inhalation of ketamine. Blood was drawn by cardiac puncture after removing the integument and peritoneum. The blood was allowed to clot at 4°C then centrifuged at 10,000 rpm for 5 min at 4°C. The supernatant pale yellow colored serum was pipetted out carefully with the help of micropipettes into fresh micro-centrifuge tubes, labeled and stored at −80°C for analysis. After the animals were sacrificed, spleen and thymus were collected aseptically. The tissues were store at −80°C until used. In each experiment, the mice were coded to ensure that the observer was blinded. Serum from different groups were normalized to the protein content by Bradford method before the assay and levels of cytokines (IL-6, IL-10, TNF-*α*, and IFN-*γ*) were determined by Sandwich ELISA according to the manufacturer's instruction in a BioRad ELISA Reader [39].

### 2.5. Reverse Transcriptase- (RT-) PCR

The total RNA was isolated using the standard TRIzol method (Gibco BRL, USA). 1 *μ*g of the total RNA was used to reverse transcribe into cDNA by One step Access RT-PCR kit (Promega, Madison, WI), followed by the amplification of the gene of interest using gene specific primers for TLR-4 (mTLR-4 sense TATCCACTGTAGCATTTCTGATATACC antisense XTCTGCTGTTTGCTCAGGATTCGAGGC) and GAPDH (Glyceraldehyde 3-phosphate dehydrogenase) [42]. PCR was performed after AMV RT inactivation and RNA/cDNA/primer denaturation for 4 minutes at 94°C, and repeating the cycles at 94°C, 55°C, and 72°C. Amplified products were separated by agarose gel electrophoresis (2%) and visualized by ethidium bromide staining. Here GAPDH is used as reference gene for quantitative real time RT-PCR. This is because certain appropriate genes are chosen and used as housekeeping genes for accurate quantitative RNA expression in real time RT-PCR technique. The expression levels of reference genes remain constant between the cells of different tissues and under different experimental conditions.

### 2.6. Quantification of C-Reactive Proteins (CRP) from Serum

Serum levels of inflammatory marker protein CRP was measured from both control and LPS treated mice by using sandwich ELISA kit, following manufacturer's guidelines (My BioSource). The minimum detectable Mouse CRP is 5 pg/mL (Intra assay Precision ≤ 8% Inter assay Precision ≤ 12%).

### 2.7. Isolation of Splenic Lymphocytes and Macrophages

Spleens were excised from killed mice and immediately placed in Alsever's solution and then macerated using frosted glass slides. Cells were repeatedly aspirated with a sterile Pasteur pipette until a single cell suspension was obtained. The suspension was then transferred to sterile tubes and kept in ice for cell debris to settle. The supernatant was then layered over 3 mL Histopaque 1077 (Sigma, USA) and then centrifuged at 1500 rpm for 30 minutes [43]. After centrifugation the band of leukocyte enriched fraction at the interface was collected and washed with DPBS, then the cell pellet was resuspended in RPMI-1640 containing 20 mM HEPES (pH 7.2) and 1 mg/mL BSA and were allowed to adhere on plastic surface for 1 hour in 37°C incubator. The nonadherent cells comprising of mix lymphocytes population were collected in a sterile centrifuge tube and centrifuged again. The pellet was resuspended in RPMI containing 10% FBS. The adherent cells comprising of macrophages, were collected by aspiration with Pasteur pipette. Cells were then washed and resuspended in culture media (RPMI + BSA) at a density of 2 × 10^6^/200 *μ*L. More than 95% cells were found viable as determined by Trypan Blue dye exclusion technique Macrophages are defined as adherent cells that ingest SRBC sensitized with rabbit IgG. About 40% of the adherent cell populations of the spleen of normal mice constitute macrophages.

### 2.8. Isolation of Thymic Lymphocytes

Thymuses were aseptically resected and placed in sterile petridishes containing RPMI-1640, penicillin 100 U/mL and streptomycin 100 U/mL. Single cell suspensions were obtained in ice-cold RPMI-1640. The suspensions were then treated with 0.1 M Tris-Hcl, pH 7.2, containing 8 g/L Tris ammonium chloride to lyse red blood cells and centrifuged at 200 ×g for 5 min at 4°C. Cell pellets were then washed three times in RPMI-1640 and resuspended in complete RPMI-1640 (containing 20 mM HEPES, 2 mM glutamine, 100 *μ*g/mL gentamicin, 100 U/mL streptomycin, and 100 mL heat inactivated FBS/Liter). Cell viability was determined by trypan blue dye exclusion. Cell suspensions were enumerated with a haemocytometer and then adjusted appropriately (2 × 10^6^/200 *μ*L) [44].

### 2.9. *In Vitro* Treatment of Lymphocytes and Macrophages with LPS

Freshly purified murine splenic macrophages and lymphocytes (2 × 10^6^/200 *μ*L) suspended in RPMI-1640 were incubated with 10 *μ*g/mL of LPS for different time periods at 37°C [45].

### 2.10. Western Blot

Western blot analysis TLR-4 expression and its modulation on LPS administration were performed by standard methods. In brief, the whole tissue was lysed with RIPA-NP40 and 60 *μ*g of the tissue lysate was separated on an 10% sodium dodecyl sulphate- (SDS-) polyacrylamide gel and blotted onto nitrocellulose membrane. The membranes were blocked with 5% BSA in TBST for 3 hour at room temperature, washed and incubated with primary anti-mice TLR-4 Abs in 1/1000 dilution (cat no- ab13867, Abcam, UK, synthetic peptide corresponding to amino acid 39–56 of mouse TLR-4) overnight at 4°C. The membranes were washed with TBST and incubated with the appropriate HRPO-conjugated secondary antibody in 1/5000 dilution (cat no-ab6721, Abcam, UK) for 1 h at room temperature. Detection of antigen was performed using the enhanced chemiluminescent detection method (ECL-plus cat no-ab140357 Abcam, UK). We have used Beta-tubulin as loading control for western blot to ensure equal loading throughout the gel as it is a housekeeping gene that exhibit high-level, constitutive expression in the sample. It also has a different molecular weight than our protein of interest that is, TLR-4, to help distinguish between both bands [14, 46].

### 2.11. Quantification of Cytokine Production from Serum and Cell Culture Supernatant

Sandwich ELISA was used to determine cytokine concentrations from serum for* in vivo* and cell culture supernatant for the* in vitro* study. We determined the levels of three major proinflammatory cytokines TNF-*α*, IL-6, and IFN-*γ* along with an anti-inflammatory cytokine IL-10 as per manufacturer's guidelines of Raybiotech, Inc, USA, in a BioRad ELISA Reader at 450 nm to establish the functionality of the expressed receptor. The minimum detectable value of TNF-*α* is <60 pg/mL, IFN-*γ* is <5 pg/mL, IL-6 <2 pg/mL, and IL-10 is <45 pg/mL. The reproducibility of cytokine kits are intra-assay: CV <10%, interassay: CV <12%.

### 2.12. Statistical Analysis

Scheffe's *F* test has been performed as post hoc test for multiple comparisons of means of different groups when significant *F* value was found. For* in vitro* study, isolated splenic macrophages, lymphocytes, and total thymic lymphocytes from mice (*n* = 6) were pooled together to obtain the requisite amount of individual cells (2 × 10^6^/200 *μ*L) and the different parameters were measured. This was repeated three times for each parameter (e.g., cytokine release in the supernatant) then the mean value of these triplicate experiments were taken for calculation. Data was expressed as mean ± S.D. Means were compared between groups by using analysis of variance (ANOVA). *P* < 0.05 was considered significant.

## 3. Results

### 3.1. Expression of TLR-4 mRNA in the Spleen and Thymus after LPS Challenge

**Figure 1:**
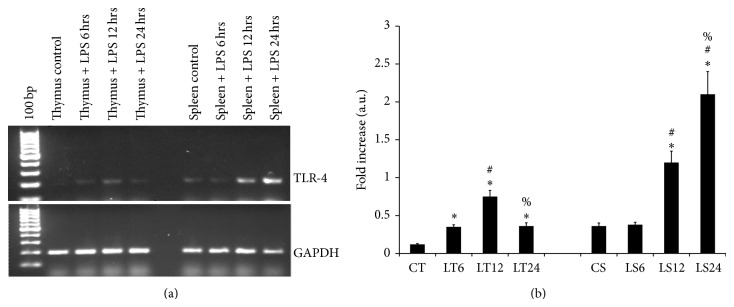
mRNA expression of TLR-4 in the spleen and thymus: the results in this figure (a) represents the differential transcription of* tlr-4 gene *on LPS administration in thymus and spleen of Swiss albino mice from triplicate experiments; lane 1 represents TLR-4 mRNA in thymus from control mice (CT), lane 2 thymus from LPS treated mice at 6 hours after treatment (LT6), lane 3 thymus from LPS treated mice at 12 hours after treatment (LT12), lane 4 thymus from LPS treated mice at 24 hours after treatment (LT24), lane 5 represents spleen from control mice (CS), lane 6 spleen from LPS treated mice at 6 hours after treatment (LS6), lane 7 spleen from LPS treated mice at 12 hours after treatment (LS12), and lane 8 spleen from LPS treated mice at 24 hours post treatment (LS24). (b) Is the diagrammatic representation of the fold difference in their expression compare to control and among different time periods of treatment. Molecular weight marker (100 bp). The following symbols indicates the significant change in the expression of TLR-4 between: ^*^control versus LPS treated tissue, ^#^LPS treatment for 6 hours versus LPS treatment for 12 and 24 hours, ^%^LPS treatment for 12 hours versus LPS treatment 24 hours. Significant change (*P* < 0.05).

Expression of TLR4 mRNA was examined in fresh tissue isolates from control and LPS challenged mice at 6 hours, 12 hours, and 24 hours after treatment. In case of thymus (Figure 1(a)) the expression of TLR-4 mRNA peaked significantly at 12 hours after treatment as compared to the untreated mice as well as to 6 hours and 24 hours of LPS treatment. On the other hand in spleen the expression of TLR-4 kept on increasing significantly with time after LPS administration at peaked at 24 hours. The change in their expression in represented in (Figure 1(b)). The relevant difference in the degree of expression between thymus and spleen is suggestive to their varied cell type as well as their function in inflammation.

### 3.2. Expression of TLR-4 Receptor Protein in the Spleen and Thymus after LPS Challenge

**Figure 2:**
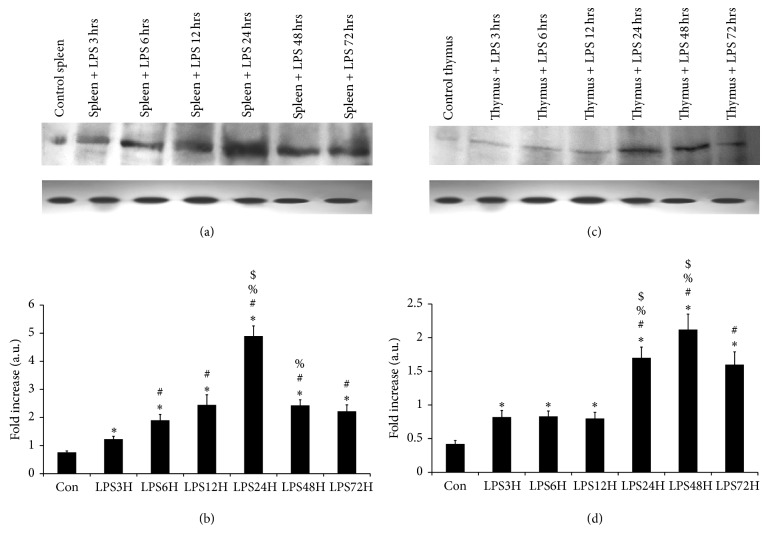
Expression of TLR-4 receptor protein in the spleen and thymus. The results in Figures (a) and (c) represent the differential translation of the TLR-4 mRNA to the protein in the spleen and thymus, respectively, in mice with experimental endotoxemia from triplicate experiments. Lane 1 (Con) represents TLR-4 in tissues from control mice, lane 2 (LPS3H) from LPS treated mice at 3 hours after treatment, lane 3 (LPS6H) from LPS treated mice at 6 hours after treatment, lane 4 (LPS12H) from LPS treated mice at 12 hours after treatment, lane 5 (LPS24H) from LPS treated mice at 24 hours after treatment, lane 6 (LPS48H) from LPS treated mice at 48 hours after treatment, and lane 7 (LPS72H) from LPS treated mice at 72 hours after treatment. Results in (b) and (d) is the diagrammatic representation of the fold difference in their expression after LPS treatment compared to control and among different time periods of treatment as seen in (a) and (c), respectively. The approximate band size is about 95 KD. The following symbols indicates the significant change in the expression of TLR-4 between: ^*^control versus LPS treated tissues, ^#^LPS treatment for 3 hours versus LPS treatment for 6, 12, 24, 48, and 72 hours, ^%^LPS treatment for 6 hours versus LPS treatment for 12, 24, 48, and 72 hours, ^$^LPS treatment for 12 hours versus LPS treatment for 24, 48, and 72 hours. Significant change (*P* < 0.05).

The receptor expression was studied at 3, 6, 12, 24, 48, and 72 hours after LPS treatment in thymus and spleen to get a wider overview of the end product. In case of thymus (Figure 2(c)) a marginal increase in the expression of TLR-4 on LPS administration was found at 3, 6, and 12 hours when compared to the control but a more noticeable increase was marked at 24 hours and 48 hours after LPS treatment not only compared to control but the previous hours of treatment as well. Then, the expression diminished dropped almost to the basal level at 72 hours after LPS injection. Comparatively in spleen (Figure 2(a)) the rise in TLR-4 expression began almost immediately after LPS treatment and a significant increase was noticed at 3, 6, and 12 hours compared to control. Almost a fourfold increase compared to control in the expression, was observed at 24 hours and then gradually the expression started decreasing on the following hours of our study. The change in their expression is represented in Figures 2(b) and 2(d), respectively.

### 3.3. Expression of TLR-4 in Isolated Splenic Macrophages, Splenic Lymphocytes, and Thymic Lymphocytes after* In Vitro* LPS Treatment

**Figure 3:**
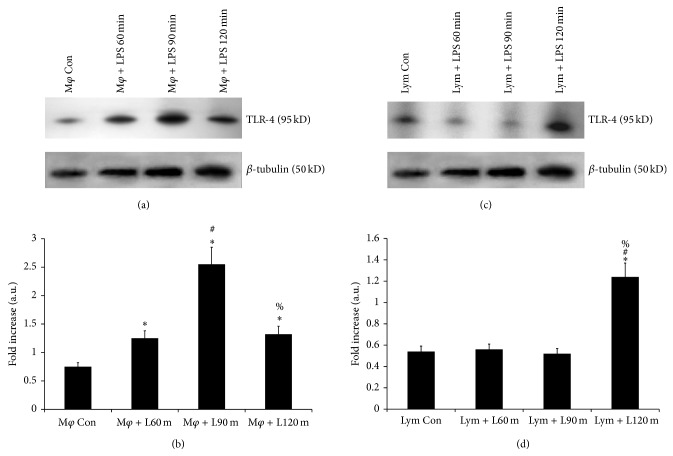
Expression of TLR-4 receptor in purified splenic macrophages and lymphocytes after* in vitro* stimulation with LPS. The results in (a) and (c) show the differential translation of the TLR-4 mRNA to the protein in the splenic macrophages and lymphocytes, respectively, after* in vitro* stimulation with LPS from a set of triplicate experiments. Lane 1 (M*φ* Con) represents TLR-4 on untreated macrophages, lane 2 (M*φ* + L60 m) represents TLR-4 expression on the macrophages treated with LPS for 60 minutes, lane 3 (M*φ* + L90 m) represents TLR-4 expression on macrophages treated with LPS for 90 minutes, and lane 4 (M*φ* + 120 m) represents TLR-4 expression on macrophages treated with LPS for 120 minutes. Lane 1 (Lym Con) represents TLR-4 on untreated lymphocyte, lane 2 (Lym + L60 m) represents TLR-4 expression on the lymphocyte treated with LPS for 60 minutes, lane 3 (Lym + L90 m) represents TLR-4 expression on lymphocyte treated with LPS for 90 minutes, and lane 4 (Lym + L120 m) represents TLR-4 expression on lymphocyte treated with LPS for 120 minutes. The following symbols represent the significant fold change in TLR-4 expression between: ^*^control versus LPS treated cells, ^#^LPS treatment for 60 min versus LPS treatment for 90 and 120 min, ^%^LPS treatment for 90 min versus LPS treatment for 120 min. Significant change (*P* < 0.05).

Expression of TLR-4 on splenic macrophages and lymphocytes was observed after LPS treatment* in vitro* for a short time interval of 60, 90, and 120 minutes. The expression on splenic macrophages increased markedly after 60 minutes of LPS treatment and peaked at 90 minutes as compared to untreated cells, but then a decrease in expression was observed after 30 more minutes (Figure 3(a)). In case splenic lymphocytes the expression was only seen to rise noticeably at 120 minutes of LPS treatment (Figure 3(c)). The change in their expression is represented in Figures 3(b) and 3(d), respectively. Expression of TLR-4 on thymic lymphocytes could not be detected on LPS treatment as the total lymphocytes isolated from untreated thymic was quantitatively not sufficient to carry out our* in vitro* model of study.

### 3.4. Quantification of Serum C-Reactive Proteins (CRP) Level

**Figure 4:**
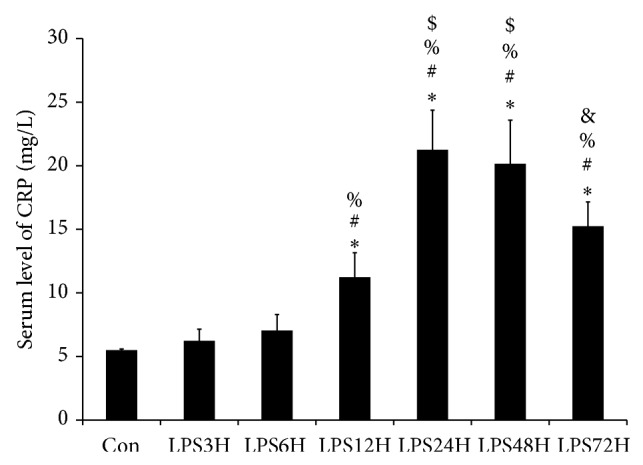
Serum CRP level in mice after experimental endotoxemia. It was observed that this inflammatory marker increased significantly at 12 to 24 hours after LPS treatment and remained high till 48 hours, indicating the onset of inflammation and the level began to decrease gradually after 72 hours. The following symbols represent the significant change between: ^*^control serum versus LPS treated serum, ^#^LPS treatment for 3 hours versus LPS treatment for 6, 12, 24, 48, and 72 hours, ^%^LPS treatment for 6 hours versus LPS treatment for 12, 24, 48, and 72 hours, ^$^LPS treatment for 12 hours versus LPS treatment for 24, 48, and 72 hours, ^&^LPS treated for 24 hours versus LPS treated for 48 and 72 hours. Significant change (*P* < 0.05). Con- control serum, LPS3H- LPS treated for 3 hours, LPS6H-LPS treated for 6 hours, LPS12H-LPS treated for 12 hours, LPS24H- LPS treated for 24 hours, LPS48H- LPS treated for 48 hours, and LPS72H-LPS treated for 72 hours.

Estimation of serum CRP levelwas done as it is an inflammatory marker and could be correlated with various cytokines secreted due to inflammation. The serum level of CRP was found to increase a few fold after 12 hours of LPS treatment then peaked at 24 hours and remained high till 48 hours after which it started to decrease (Figure 4). This was suggestive as unlike human, CRP is not a truly acute phase response protein in mice as reported in other strains earlier.

### 3.5. Measurement of Serum Tumor Necrosis Factor *α* (TNF-*α*), Interleukin-6 (IL-6), Interferon Gamma (IFN-*γ*), and Interleukin-10 (IL-10)

**Figure 5:**
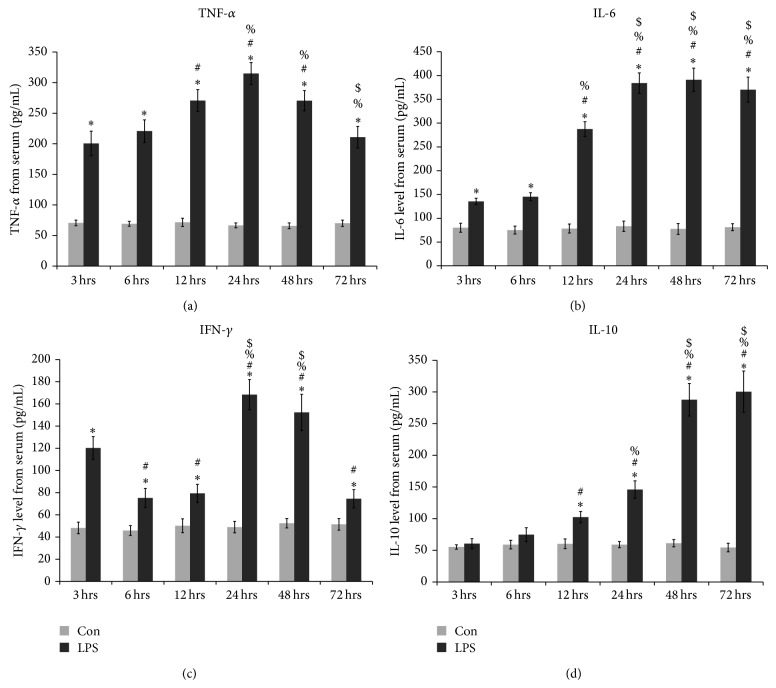
Serum cytokine levels in Swiss albino mice after experimental endotoxemia. Serum levels of (a) TNF-*α*; (b) IL-6; (c) IFN-*γ*; and (d) IL-10 in control and LPS treated mice at 3, 6, 12, 24, 48, and 72 hours after treatment showed time-dependent variation with the onset of endotoxemia (values are expressed as Mean ± SD from triplicate experiments and are significant (*P* < 0.05) from 6 mice in each group). The following symbols represent the significant difference between: ^*^control versus LPS treated serum, ^#^LPS treatment for 3 hours versus LPS treatment for 6, 12, 24, 48, and 72 hours, ^%^LPS treatment for 6 hours versus LPS treatment for 12, 24, 48, and 72 hours, ^$^LPS treatment for 12 hours versus LPS treatment for 24, 48, and 72 hours.

Quantitative analysis of some proinflammatory and anti-inflammatory serum cytokine was done to attest the functionality of TLR-4 and to examine its regulatory role in inflammation in our* in vivo *model of endotoxin induced inflammation. We examined proinflammatory cytokines like TNF-*α*, IFN-*γ*, and IL-6. Serum IL-10 level was measured, being the most important and widely studied anti-inflammatory cytokine so far. The analysis was done at 3, 6, 12, 24, 48, and 72 hours after LPS challenge. The serum level of TNF-*α* increased with time from the earlier hours of LPS treatment and peaked at 24 hours and then gradually started decreased in the next period of study (Figure 5(a)). The serum level of IL-6 was found to rise slowly after treatment and it peaked at 24 hours but sustained at that level in the later hours of the study (Figure 5(b)). The secretion of IFN-*γ* was irregular initially, raised at 3 hours post treatment and then returned to the basal level at 6 and 12 hours. Again increased significantly at 24 hours compared to normal and the earlier hours of treatment but started to return to normal in the 48th hour of our study and returned to normal at 72nd hour (Figure 5(c)). The serum level of anti-inflammatory cytokine IL-10 started rising in the late hours of LPS treatment and kept on increasing even at 72 hours of treatment (Figure 5(d)).

### 3.6. Quantification of Tumor Necrosis Factor *α*, Interferon Gamma, Interleukin-6, and Interleukin-10 Released in the Media after* In Vitro* LPS Stimulation of Splenic Macrophages

**Figure 6:**
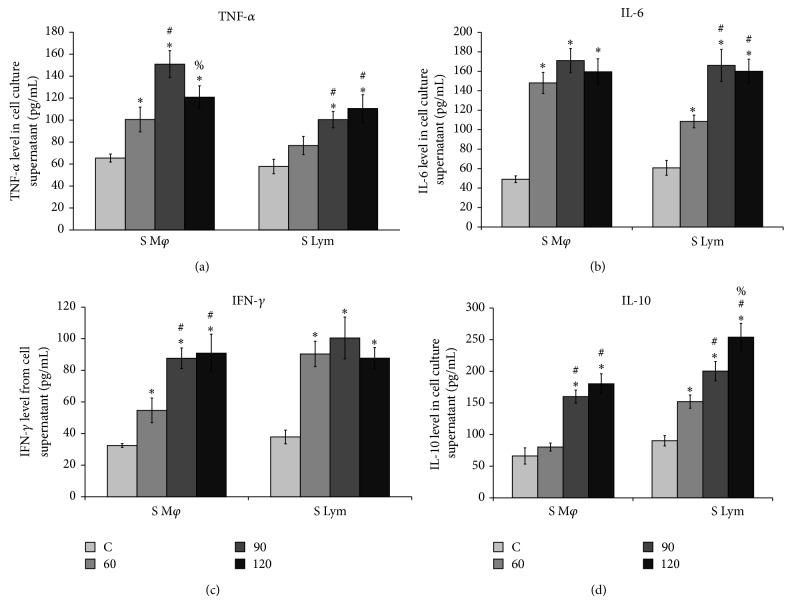
Analysis of cytokines from the cell culture supernatant of splenic macrophages and lymphocytes after* in vitro* LPS stimulation. Levels of (a) TNF-*α*; (b) IL-6; (c) IFN-*γ*; and (d) IL-10 in the supernatant of untreated and LPS stimulated splenic macrophages at 60, 90, and 120 minutes after treatment showed time-dependent cytokine production after endotoxin stimulation (values are expressed as Mean ± SD from triplicate experiments and are significant (*P* < 0.05) from 6 mice in each group). The following symbols represent significant difference between: ^*^control versus LPS treated cells, ^#^LPS treatment for 60 minutes versus LPS treatment for 90 and 120 minutes, ^%^LPS treatment for 90 minutes versus LPS treatment for 120.

Measurement of the above mentioned cytokines from the cell culture supernatant was done after 60, 90, and 120 minutes of LPS treatment. The level of TNF-*α* in the cell supernatant of splenic macrophages remained significantly higher on LPS treatment throughout as compared to the untreated cells. The peak was noticed after 90 minutes of treatment but a decrease was marked after 120 minutes. In case of lymphocytes the difference in the level of TNF-*α* increased only marginally with time (Figure 6(a)). In case of IL-6 it was seen that a significant rise reached in the first hour from the splenic macrophage supernatant treated with LPS but no noticeable change was observed in the later hours, but in the supernatant from the treated lymphocyte population the highest concentration reached at 90 minutes post treatment (Figure 6(b)). The level of IFN-*γ* increased from just detectable levels to almost double the value in the first hour and then increased with time till 120 minutes. In case of lymphocytes the peak reached after 90 minutes after treatment and then a slight decrease was observed (Figure 6(c)). A correlation was rather observed in the IL-10 level between* in vivo* and* in vitro* study, as in both cases a late rise in its level was observed (Figure 6(d)) after treatment both in the macrophages and lymphocytes.

## 4. Discussion

TLRs are the mammalian homologues of the toll receptors of* Drosophila* and are known to be key regulators of innate immune responses [47]. They are cell surface receptors that play a cardinal role in the recognition of pathogen-associated molecular patterns (PAMPs) [48] and through them the host can distinguish between various pathogens and can induce the appropriate responses. Until now, 11 different members of the TLR family have been identified in mammals and in this study TLR-4 was focused due to its established role in innate immunity against Gram-negative bacterial component. TLR-4 could recognize lipopolysaccharide of Gram-negative bacteria and contribute in the host defense against them by eliciting immune response [49]. The differential expression of TLR-4 might occur through a variety of mechanisms which reflect the adaptation of the innate immune system depending on the different anatomical localization and different microbial milieu encountered at each anatomical location. Characterization of TLR-4 in the lymphoid organs and cells were done in order to provide a basic idea about their role in early endotoxemia in this unreported mouse model, so that the primary stage of clinical studies could be planned and initiated, considering TLR-4 and its associated signaling pathway/molecules as therapeutic targets.

The mammalian immune system aspire cooperation between the innate and acquired wings providing an optimal environment for defense against the invasion of pathogens at any site in the body. The locales of organized lymphoid cell accumulations are known as primary and secondary lymphoid organs [50] and in the current study, constitutive* in vivo* expression of TLR-4 in the lymphoid organs and their isolated cell subtypes of Swiss albino mice was observed to discern its modulation by* E. coli* LPS. The expression of TLR-4 in spleen was observed, which is the largest secondary lymphoid organ and was compared with thymus, the primary one. The aim was to identify the initial changes in its expression when the body encountered LPS for a short time, because although lymphoid organs are located in anatomically distinct sites but dynamism of lymphoid organs has been established, especially in the secondary ones which are quite plastic and influenced by their environment [51]. For the* in vitro* study splenic macrophages were isolated which has been considered as representative of macrophages in endotoxin studies, because peritoneal macrophages were found to perish by cytocidal effect of endotoxin-associated ascitic fluid. The second type of cell isolated was lymphocytes as TLRs are thought to regulate adaptive immunity indirectly through the activation of innate responses but some reports suggested that they could modulate the function of lymphocytes as well [52–54]. In the* in vivo* system lymphocytes also produce proinflammatory cytokines in response to an antigenic stimulation. In order to clarify whether TLR-4 expression on the lymphocytes are enhanced after LPS treatment for a short time and have a role in proinflammatory cytokine production, individual studies on both these types of cells were commenced.

The expression of TLR-4 was demonstrated at mRNA level in spleen and thymus up to 24 hours after LPS challenge and its expression pattern was found to grossly coincide with that of the end product in spleen, whereas a variation was observed in thymus, ratifying the presence of any post transcriptional modification or any other factors on LPS administration in thymus. The intensity of TLR-4 expression on spleen was much greater as compared to thymus which could be due to greater number of macrophages and lymphocytes encountering antigen at a time [55, 56]. However, the time-dependent increase in the expression of TLR-4 and then downregulation at the later hours cannot be explained from these findings and require a more detail study at molecular level.

In case of receptor protein in the* in vivo* system, the expression of TLR-4 was monitored at time periods of 3, 6, 12, 24, 48, and 72 hours after endotoxin administration to get a much wider overview of the expression pattern and difference in the onset of upregulation of TLR-4 expression among the two organs was observed. Moreover, in case of thymus the peak in mRNA expression was observed after 12 hours of LPS treatment but that of the protein was observed after 24 hours of LPS treatment speculating some factors at post transcriptional or translational level responsible for this delay.

While stimulating the splenic cells* in vitro* a much shorter time period was considered compared to the* in vivo* study. Thus, because, although from the* in vivo* study it was found that 24 hours of LPS stimulation could be considered to be a reasonable time frame for cytokine production dependent on gene transcription, but whether any additional advantage could be provided to the cells by allowing shorter period of LPS stimulation was required to be determined. The expression of TLR-4 on macrophages was diminished after 120 minutes of LPS challenge accompanied with the depression of cytokine production, indicating the suppression of innate immune system. Detection of TLR-4 expression in the splenic lymphocytes on short term LPS stimulation was not possible in the present study so a detailed study on the lymphocytes perhaps by more sensitive methods like FACS and immunostaining could be performed in future to get an overview of their expression and modulation on LPS stimulation.

LPS sepsis is noninfectious (sterile) hence participation of cytokines is highly suggestive for this inflammation in the early stage. Some studies have shown that macrophage migration inhibitory factor, interferon-gamma, and IL-2 upregulate the expression of TLRs [57] and that colony-stimulating factor and IL-4 downregulate the expression [58].

In order to characterize the functional relevance of TLR-4 in cells and tissue, the production of the proinflammatory as well as anti-inflammatory cytokines in response to LPS was determined. Serum of the experimental mice challenged with LPS are supposed to reflect the primary site of inflammation for the* in vivo* study and the cell culture supernatant in the* in vitro* study [59]. It has been generally reported that TLRs are upregulated in inflammatory conditions [21] and downregulated in immunosuppressive conditions [20]. Therefore, also in LPS endotoxemia, it could be postulated that the upregulation of TLRs may facilitate the inflammatory response (reflected by the levels of TNF-*α*, IL-6, IFN-*γ*, and IL-10) and function protectively against infection whereas the downregulation of TLRs may suppress the inflammation and facilitate the subsequent infection.

The release of acute phase proteins and corticosterone is a major feature of acute phase reactions and many of acute phase proteins are antiprotease inhibitors in nature. The main function of acute phase reactions could be regarded as part of anti-inflammatory mechanisms generally mediated by IL-6. It was observed in this study that inflammatory marker CRP level peaked from 12 to 24 hours indicating endotoxin induced inflammation, and the sustained high levels of CRP could be due to constant stimulation from IL-6 in the host body. It has been established in previous works that inflammatory responses elicited by endotoxin, inhibition of proinflammatory cytokines, induction of anti-inflammatory molecules and extra hepatic protease inhibitors is generally operated by IL-6 thus regulating the extent of tissue inflammatory responses [60, 61].

It could be concluded from the present study that Swiss albino strain of mice are responsive to LPS stimulation and* tlr-4 gene* is transcribed to mRNA and then translated to TLR-4 receptor protein in a time-dependent manner and the expression pattern varied among the lymphoid organs and their cell populations. This upregulation of receptor expression was accompanied by increase in proinflammatory cytokines and in the later hours by anti-inflammatory cytokine. This result is in contrast to prior reports on C3H/HEJ mice in which TLR-4 had been characterized in details and found that they were unresponsive to LPS due to mutation in* tlr-4 gene*. It was also reported that production of proinflammatory cytokines like TNF-*α* and IFN-*γ* was impaired compare to wild type strain [11]. The expression of TLR-4 after LPS stimulation in Swiss albino mice is comparable with other strains like C57/BL6 and HAM/ICR (CD-1) which were reported to be highly responsive to LPS leading to upregulation of TLR-4 along with TNF-*α* [62, 63]. Other than mice strains B16 tumor cell line has also been studied extensively in regard to TLR-4 and showed that IFN-*γ* was the critical factor produced by TLR-4 activated tumor cells in mediating* in vitro* outgrowths and is enhanced after LPS stimulation [64]. Although the report was in a completely different cell line, Swiss albino mice showed similar increase in IFN-*γ* production which could contribute in the aggravation of endotoxemia as well.

As literature suggested, the expression of TLR-4 is modulated by a variety of environmental factors, such as microbial invasion, microbial components, and cytokines in different organs and cells [65] but no information was available regarding the influence of LPS on TLR-4 expression in Swiss albino mice. This study showed that TLR-4 receptor is expressed in both primary and secondary types of lymphoid organs of Swiss albino mice, but its time and rate of modulation differed in LPS induced endotoxemia, which could give an insight of the divergent functional activity of their distinct cell population in inflammation. The sustained higher levels of cytokines IL-6 and IL-10 at the later hours could be partially responsible for imparting a protective role in endotoxemia. Pretreatment with IL-6 or IL-10 could provide a protection in endotoxemia by inhibiting tissue degradation as it has already been established in our prior studies that pretreatment with IL-6 lowers liver damage in experimental endotoxemia by modulating ROS production and cell infiltration [66]. It could also stimulate IL-10 which in turn could suppress proinflammatory cytokines due to their immune response-limiting properties. However, an inflammatory response with excessive production of proinflammatory cytokines as seen in the early hours could induce side effects depending on the intensity of the disease progression and could even lead to multiple organ dysfunction syndrome and death. So therapeutic studies with antibiotics alone or in combination are suggested in this model for application in the clinical field. Another approach could be a tight regulation of TLR-4 signaling such as receptor blocking to prevent unwanted or prolonged stimulation which might be harmful for the host.

## Abbreviations

BSA: Bovine serum albumin
CD14: Cluster of differentiation 14
CPCSEA: Committee for the Purpose of Control and Supervision of Experiments on Animals (India)
CRP: C-reactive protein
DPBS: Dulbecco's phosphate-buffered saline
ELISA: Enzyme linked immunosorbent assay
FBS: Fetal bovine serum
GAPDH: Glyceraldehyde 3-phosphate dehydrogenase
HEPES: Hydroxyethyl piperazineethanesulfonic acid
IAEC: Institutional Animal Ethical Committee
IFN-*γ*: gamma interferon
IL-1: Interleukin 1
IL1R1: Interleukin 1 receptor type 1
iNOS: Inducible nitric oxide synthase
IP: IFN-*γ*-inducible protein
IRF-3: Interferon regulatory factor 3
LBP: LPS-binding protein
LPS: Lipopolysaccharide
LTA: Lipoteichoic acid
MAPK: Mitogen-activated protein kinases
MIF: Macrophage inhibitory factors
MYD88: Myeloid differentiation primary response 88
NF-*κ*B: nuclear factor-*κ*B
PAMP: Pathogen-associated molecular pattern
RIPA: Radioimmunoprecipitation assay buffer
RNI: Reactive nitrogen intermediates
ROS: Reactive oxygen species
RPMI: Roswell Park Memorial Institute medium
SAP: Serum amyloid P-component
SDS-PAGE: Sodium dodecyl sulphate polyacrylamide gel electrophoresis
TLR: Toll-like receptor
TNFR: Tumor necrosis factor alpha receptor
TNF-*α*: Tumor necrosis factor alpha-*α*
TRAF 6: TNF receptor associated factor.
